# Classroom environment and learning engagement in mental health education courses: prospective indirect associations through two dimensions of grit among first-year students in East China

**DOI:** 10.3389/fpsyg.2026.1927030

**Published:** 2026-10-02

**Authors:** Weijun Wang, Heng Hu, Chen Cai

**Affiliations:** 1 School of Chenyang, Zhejiang Shuren University, Hangzhou, China; 2 Humanities and Foreign Languages, Zhejiang Shuren University, Hangzhou, China

**Keywords:** classroom environment, consistency of interest, grit, learning engagement, mental health education courses (MHEC)

## Abstract

This study examines prospective associations among classroom environment, perseverance of effort (POE), consistency of interest (COI), and learning engagement in mental health education courses (MHEC). Participants were 468 first-year students from three schools at a university in East China. A three-wave time-lagged survey measured classroom environment at T1, the two grit dimensions at T2, and learning engagement at T3. Because each construct was measured only once and baseline levels were not controlled, the design establishes temporal separation but does not constitute a fully longitudinal mediation design or support causal inference. Structural equation modeling indicated that classroom environment and POE were positively associated with learning engagement, whereas the unique association between COI and learning engagement was not statistically significant. Bias-corrected bootstrap analysis showed a significant prospective indirect association through POE, but not through COI. Because the two specific indirect effects were not formally contrasted, the findings should be interpreted as preliminary evidence of dimension-specific patterns rather than evidence that the two indirect pathways differ significantly.

## 1 Introduction

As mental health issues and disorders among youth continue to pose public health challenges, mental health professionals and educators have paid growing attention to mental health literacy (McLuckie et al., 2014). Research shows that Mental Health Education Courses (MHEC) can strengthen students’ psychological resilience and help them regulate emotions when facing academic and life stress (McConville et al., 2017). In Chinese higher education, MHEC are part of holistic education. They aim to impart psychological knowledge, cultivate positive traits, and support personal development (Gao and Zong, 2017). However, Chinese universities face challenges in implementing MHEC, including low student motivation and limited classroom engagement, which hinder educational goals and psychological growth (Li, 2024). The theory of learning engagement posits a positive correlation between students’ classroom engagement and their learning outcomes and instructional effectiveness (Hendrick et al., 2023). In MHEC, engagement is crucial because psychological competence and emotional management require active participation and deep internalization (Davies et al., 2018). Understanding the factors associated with student engagement in MHEC is therefore an urgent concern for researchers and practitioners.

Given that classroom environments are the primary setting for mental health education, existing research has mainly examined how environmental factors relate to university students’ learning engagement (Huang and Xie, 2023). Classroom climate, teacher-student interactions, and instructional resources are associated with motivation and participation (Wang et al., 2023). However, environmental factors alone do not fully explain variation in engagement (Halverson and Graham, 2019). Grit is a relatively stable but domain-sensitive disposition that includes perseverance of effort and consistency of interest. It may help students overcome learning difficulties and sustain motivation, which may relate to learning engagement (Credé et al., 2017). However, the two dimensions may function differently across domains and course contexts (Clark and Malecki, 2019; Credé et al., 2017; Teimouri et al., 2022). MHEC are compulsory and oriented toward value internalization (Gao and Zong, 2017; Tan and Ma, 2020). Students may enter with low or unstable interest. In this context, interest is not a stable precondition but a developmental outcome. Value internalization requires sustained effort, especially when concepts are complex or personally challenging. Perseverance of effort may therefore be more central in MHEC, whereas consistency of interest may be less predictive when interest is still forming. Because personality traits are complex, the mechanisms linking grit to learning engagement remain inadequately understood.

MacIntyre’s (2016) Three Pillars Theory of Positive Psychology suggests that learning outcomes arise from personality traits, positive emotions, and environmental factors. However, this theory does not specify a temporally ordered process. This study therefore integrates Self-Determination Theory (Ryan and Deci, 2000) and Social Cognitive Theory (Bandura, 2002). Self-Determination Theory (SDT) explains how classroom environment may support or frustrate autonomy, competence, and relatedness, which are relevant to sustained effort and interest. Social Cognitive Theory (SCT) explains how environmental feedback and individual characteristics interact to guide learning behavior. Together, these theories motivate a temporally ordered model in which classroom environment precedes grit and learning engagement. Given the present measurement schedule, this model is interpreted as a prospective indirect-association model rather than as evidence of causal or change-based mediation.

It addresses three questions. (1) Is classroom environment prospectively associated with learning engagement in MHEC? (2) Is perseverance of effort positively associated with learning engagement, and what is the unique association between consistency of interest and learning engagement? (3) Do the two grit dimensions carry prospective indirect associations from classroom environment to later learning engagement? Most existing studies are single-wave and cross-sectional, leaving temporal ordering unclear. The three-wave time-lagged survey improves temporal separation by measuring T1 classroom environment, T2 grit, and T3 learning engagement in sequence. However, because baseline levels of the constructs were not measured and each construct was assessed only once, the design cannot estimate within-person change, autoregressive effects, or a fully longitudinal mediation process. By separating the two grit dimensions, the study avoids masking potentially different associations. The findings apply to first-year students from three schools at one university in East China and should not be generalized to all Chinese university students.

## 2 Literature review

### 2.1 Learning engagement

Learning engagement refers to the extent to which students maintain focus and actively participate in the learning process and the completion of tasks (Skinner et al., 2009). It is a multi-faceted construct, incorporating three primary components: behavioral engagement, emotional engagement, and cognitive engagement. These elements constitute the foundational aspects of the concept (Ren, 2023). Behavioral engagement is reflected in students’ participation in class activities and their completion of tasks. Emotional engagement is defined by students’ interest and emotional investment in the learning process, while cognitive engagement pertains to the depth of students’ cognitive involvement and their capacity to assimilate knowledge (Fredricks et al., 2004). Empirical studies have demonstrated that learning engagement is a significant predictor of academic success (Pang and Veloo, 2024) and is strongly linked to psychological well-being and social adaptability (Strobel, 2024). Students with high levels of engagement typically show greater psychological resilience, reduced anxiety, and stronger social integration (Hu and Xiao, 2025). Consequently, learning engagement is commonly regarded as an essential element for both academic success and the capacity to adapt within higher education.

The factors influencing learning engagement are complex and function across various levels, involving both personal and environmental elements. At the individual level, Hanrahan et al. (2018) underscore intrinsic motivation as a primary driving force behind heightened engagement, arguing that when students develop genuine interest in or identification with learning content, their level of engagement significantly increases. On the environmental level, factors like the classroom atmosphere, interactions between teachers and students, and relationships among peers are crucial in shaping the learning experience. Mukherjee et al. (2024) find that positive peer interactions facilitate engagement by providing social support and learning resources, whereas negative peer interactions may trigger anxiety and alienation, thereby inhibiting engagement. Additionally, the supportiveness of the classroom environment, diversity in teaching methods, and the practical relevance of course content have been empirically validated as crucial environmental determinants of engagement (Wei et al., 2019). However, existing research remains insufficient in explaining how environmental and individual factors interact to shape learning engagement.

Furthermore, studies on learning engagement have predominantly focused on general education courses, with limited research on its application in specific academic domains. Within the context of MHEC, how to understand and predict students’ engagement remains underexplored. A distinctive feature of MHEC lies in their dual pedagogical objectives: beyond teaching theoretical knowledge, they necessitate students’ internalization of psychological concepts into practical competencies such as emotional regulation and stress management. This inherent complexity imposes higher demands on students’ engagement. Consequently, existing learning engagement theories require further empirical validation within the specific pedagogical framework of mental health education to clarify their applicability and effectiveness in this specialized educational context.

### 2.2 Classroom environment and learning engagement

The classroom environment refers to the various physical, psychological, and social settings in which students engage in their educational activities. This environment includes interactions between teachers and students, relationships among peers, and the general mood or atmosphere within the classroom (Dorman et al., 2006). Reyes et al. (2012) suggest that when educators employ supportive teaching strategies, students are more likely to exhibit positive behaviors and attitudes toward learning. Similarly, Lai (2021) highlights the significant role of constructive peer interactions in enhancing both learning engagement and academic success. Collectively, these findings underscore the crucial impact of a nurturing and positive classroom atmosphere on fostering student learning engagement.

However, the beneficial effects of the classroom environment on learning engagement are not absolute. Gao (2016) finds that students’ responses to the same environment can vary significantly, with some individuals thriving in high-pressure settings while others exhibit greater engagement in more relaxed atmospheres. Joe et al. (2017) further contend that the classroom environment does not directly predict learning engagement but rather may be indirectly associated through psychological needs, learning motivation, and emotional states. These contradictory findings suggest that the relationship between classroom environment and learning engagement may be more complex than previously assumed, potentially involving mediating or moderating mechanisms.

The current study examines three key aspects of the classroom environment: teacher immediacy, group cohesion, and task orientation, each aligning with the essential components of classroom instruction: the teacher, the students, and the learning material (Peng and Woodrow, 2010). Teacher immediacy creates a sense of security and offers professional guidance, while group cohesion encourages mutual support and collaboration among peers. Meanwhile, varied learning tasks promote hands-on, experiential learning (Tan and Ma, 2020). The interaction of these elements collectively forms a classroom environment that supports students’ psychological well-being. By exploring the relationship between classroom environment and learning engagement within the context of MHEC, this study aims to enhance understanding of their intricate connection. Based on this, the following research hypothesis is proposed:

*H1*: The classroom environment is positively associated with learning engagement.

### 2.3 Grit and learning engagement

Grit refers to a person’s determination and dedication to achieving long-term objectives, encompassing two key components: perseverance of effort and consistency of interest (Duckworth et al., 2007). Perseverance of effort refers to the tendency to maintain sustained effort despite difficulties, whereas consistency of interest reflects the stability of one’s passions over time (Duckworth and Quinn, 2009). Although these two dimensions are related, recent research suggests that they may have distinct associations with learning outcomes, particularly in domain-specific contexts (Clark and Malecki, 2019; Teimouri et al., 2022). Dixson (2021) argues that grit is just as crucial as inherent ability in determining students’ academic success. Due to its significant role in academic performance, researchers have sought to incorporate the concept of grit into educational studies (Teimouri et al., 2022). Nonetheless, the relationship between grit and learning engagement is not well-defined in the current body of research. Datu et al. (2018) suggest that students who exhibit higher levels of grit tend to sustain their attention and consistently invest effort in academic tasks, resulting in improved academic performance. In contrast, other research highlights the context-dependent nature of grit (Paradowski and Jelińska, 2024), suggesting that its relationship with learning engagement differs depending on the learning environment. Furthermore, Credé et al. (2017) warn that, in certain situations, excessive grit can lead to burnout, causing increased academic stress and fatigue.

MHEC frequently address complex and sensitive social, political, and ethical issues, which not only challenge students’ cognitive abilities but may also evoke emotional fluctuations and ethical dilemmas. Students might question culturally specific standards of mental health or engage in critical discussions about the stigmatization of psychological disorders, thereby prompting deeper reflections on social justice and moral reasoning (Tan and Ma, 2020). When confronted with perspectives that diverge from their preexisting beliefs, students may experience cognitive dissonance and psychological stress. In this context, grit may play a particularly crucial role in sustaining a positive learning attitude and adaptive learning behaviors in mental health learning. Because MHEC are compulsory and oriented toward value internalization, students may enter with low or unstable interest. Consistency of interest assumes a stable pre-existing interest, whereas value internalization requires sustained effort when topics are sensitive, cognitively challenging, or emotionally activating. Therefore, perseverance of effort may be more relevant than consistency of interest in this context.

Building on the two-dimensional framework of grit, this study examines POE and COI separately. Prior evidence provides a relatively clear basis for expecting POE to be positively associated with engagement because sustained effort is directly relevant to continued participation under difficulty (Datu et al., 2018; Teimouri et al., 2022). The prediction for COI is less certain. COI may be positively related to engagement when stable interest is already present, but in compulsory MHEC students may enter with low or developing interest, making a directional prediction less defensible (Credé et al., 2017; Clark and Malecki, 2019). Accordingly, a directional hypothesis is retained for POE, whereas the unique association of COI with learning engagement is examined as a research question:

*H2*: Perseverance of effort is positively associated with learning engagement.

RQ1: What is the unique association between consistency of interest and learning engagement in MHEC after accounting for the other variables in the model?

### 2.4 Classroom environment, grit, and learning engagement

The Three Pillars Theory of Positive Psychology offers a broad map of positive functioning (MacIntyre, 2016). Its value here lies in linking classroom conditions, grit, and engagement within one framework. SDT and SCT further specify motivational and social-cognitive processes through which an earlier classroom environment may be associated with later learning behavior. The present study uses these theories to motivate a temporally ordered prospective indirect-effects model from T1 classroom environment to T2 grit dimensions and T3 learning engagement. Because baseline levels were not controlled and the same constructs were not repeatedly measured, this temporal ordering should not be interpreted as a causal longitudinal mediation process.

The two a paths also require theoretical justification. Teacher immediacy offers security, guidance, and feedback and may strengthen competence and relatedness, thereby supporting perseverance of effort (Ding and Wang, 2025). Group cohesion provides peer support and belonging and may sustain both effort and interest (Yoon et al., 2025). Task orientation connects course content to students’ experience and may support situational interest (Dao, 2021). However, in compulsory MHEC, stable interest may not be present at course entry and can instead develop through participation. For this reason, the pathway involving POE is specified directionally, whereas the role of COI is treated as theoretically less certain and is examined without an unequivocal positive prediction.

The two grit dimensions also require separate treatment. A composite grit score mixes perseverance of effort and consistency of interest. If the two dimensions relate to engagement in different ways, the composite score may hide or offset these associations (Clark and Malecki, 2019). In MHEC, value internalization demands sustained effort when topics are sensitive, cognitively demanding, or emotionally activating (Gao and Zong, 2017; Tan and Ma, 2020). Consistency of interest assumes that stable interest already exists. This assumption fits elective courses better than compulsory ones. For compulsory MHEC, perseverance of effort is the more relevant dimension for explaining continued engagement.

Most prior studies on grit and engagement are cross-sectional and single-wave. A three-wave time-lagged design improves temporal separation by placing T1 classroom environment before T2 grit and T3 learning engagement. Nevertheless, temporal separation alone does not establish longitudinal mediation because each construct was measured only once and prior levels were not controlled. Accordingly, the present analyses test prospective indirect associations rather than change in grit or causal mediation. In this study, grit is treated as a domain-sensitive disposition in the MHEC context, not as a fixed global personality trait that is assumed to change within 6 weeks. One directional hypothesis and one research question are proposed:

*H3*: Classroom environment has a positive prospective indirect association with learning engagement through perseverance of effort.

RQ2: Does consistency of interest carry a prospective indirect association between classroom environment and learning engagement?

### 2.5 Prospective indirect-effects model

This study specifies a parallel prospective indirect-effects model with three temporally separated measurement occasions. T1 classroom environment is the predictor, T2 perseverance of effort and consistency of interest are parallel intervening variables, and T3 learning engagement is the outcome. The model also includes a direct path from T1 classroom environment to T3 learning engagement. Classroom environment and learning engagement are modeled as second-order latent variables with their dimension scores as first-order factors and items as observed indicators, whereas POE and COI are modeled as latent variables with their four respective items as observed indicators. The model tests whether the products of the temporally ordered paths are different from zero; it does not estimate within-person change or control prior levels of the mediators or outcome. Because a formal contrast between the two specific indirect effects was not available in the present analysis, no claim is made that the two indirect effects differ statistically. Figure 1 presents the model.

**Figure 1:**
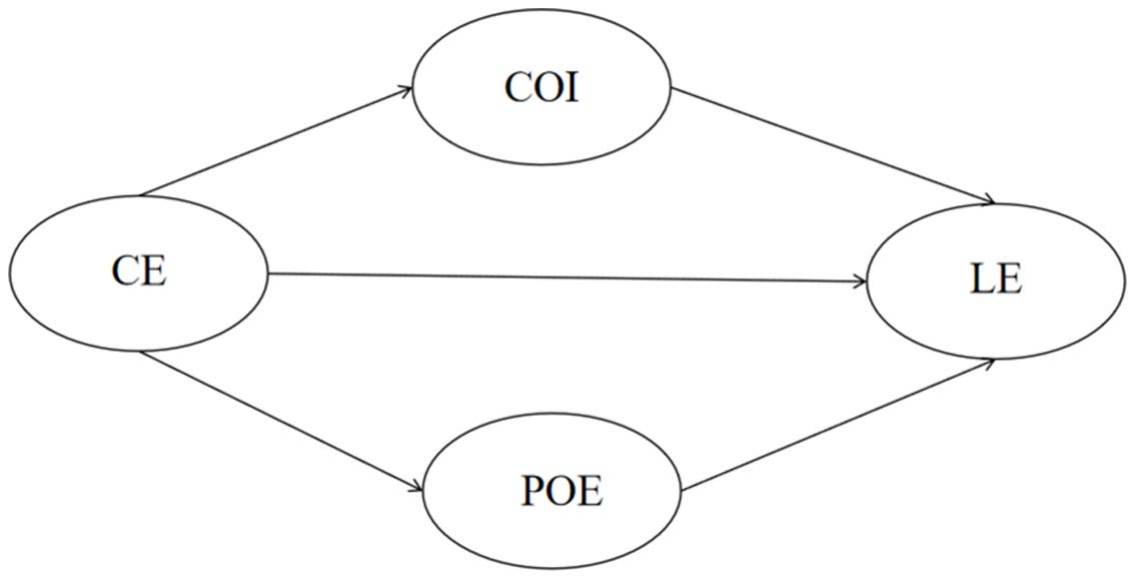
Theoretical framework.

## 3 Research design

### 3.1 Participants

This study was approved by the Ethics Committee of Zhejiang Shuren University (Approval No. SR-2023-0416; approved in August 2023). A convenience sampling method was employed, involving 550 first-year students from three schools at a university in East China. The three schools were selected based on convenience and accessibility, and instructors invited students from intact classes to participate voluntarily. No students declined participation. At the time of the survey, all participants had enrolled in the Mental Health Education Course (MHEC), which spanned 17 weeks (September 2023 to January 2024).

All surveys were administered via the online platform Wenjuanxing.^1^ This study used a three-wave time-lagged survey design. Classroom environment was measured at Week 9 (T1), grit at Week 15 (T2), and learning engagement at Week 17 (T3). Each construct was measured only once. Therefore, the design establishes temporal separation among measurements but does not control baseline levels, estimate autoregressive change, or provide strong longitudinal mediation or causal evidence (Sürücü et al., 2023).

Following rigorous data review, 82 incomplete responses (14.91%) were excluded due to missing key information or inconsistencies across the three waves. Consequently, the final dataset comprised 468 valid responses (students who completed all three waves), yielding a valid response rate of 85.09%. An attrition analysis comparing the 468 completers with the 82 non-completers on gender, School, and T1 classroom environment showed no significant differences (all *p* > 0.05). A Monte Carlo power analysis for indirect effects based on the 32-indicator structural model indicated that with *N* = 468, the study had 80% power to detect a small indirect effect (*β* = 0.08). Students were nested within 12 classes taught by 4 instructors. Intra-class correlations ranged from 0.03 to 0.06, indicating minimal clustering. Cluster-robust standard errors were also examined as a robustness check. Participants’ mean age was 19.72 years (SD = 1.62). Table 1 presents a summary of the demographic characteristics.

**Table 1:** Summary of the sociodemographic information of the participants.

Sociodemographic variables	Frequency	Percent
Gender		
Male	271	57.91%
Female	197	42.09%
School		
School of Humanities	173	36.96%
School of Architecture	131	27.99%
School of Information	164	35.02%
Total	468	100%

### 3.2 Research tools

All three questionnaires were originally developed in English. Following standard translation and back-translation procedures (Narasimhan and Yashaswini, 2025), two bilingual researchers independently translated the scales into Chinese. After reconciling discrepancies, a third translator blind to the original versions back-translated the Chinese version into English. The back-translated version was compared with the original, and minor wording adjustments were made to ensure conceptual equivalence. A panel of three experts (two in educational psychology and one in psychometrics) reviewed the Chinese items for content validity. Minor context-specific adaptations were made, and prior Chinese validation evidence for the scales was considered. A pilot test with 30 Chinese university students (not included in the final sample) confirmed the clarity and comprehensibility of the Chinese items.

#### 3.2.1 Learning engagement scale

This study employed the *Learning Engagement Scale* developed by Fredricks et al. (2005) and validated by Liao (2011) to evaluate Chinese college students’ learning engagement. It comprises three dimensions: behavioral engagement, emotional engagement, and cognitive engagement, with a total of 11 items. Behavioral engagement pertains to learners’ observable behaviors and their level of participation in learning activities, consisting of four items. An example item is: I *always complete the assignments given by the teacher on time*. Emotional engagement refers to the positive affective experiences learners undergo during the learning process and also comprises four items, such as: *I feel relaxed in class*. Cognitive engagement captures the extent of cognitive effort learners are willing to exert to comprehend and master knowledge and skills, represented by three items. An illustrative item is: *I attempt various approaches to overcome learning difficulties*.

Each item was assessed using a 5-point Likert scale, where 1 represented strongly disagree and 5 represented strongly agree. In the present sample, the scale demonstrated acceptable model fit indices (χ^2^/df = 1.462, CFI = 0.981, TLI = 0.983, RMSEA = 0.037, SRMR = 0.031) and good internal consistency (Cronbach’s α = 0.895). LE was modeled as a second-order latent factor, with behavioral, emotional, and cognitive engagement as first-order factors and their respective items as observed indicators.

#### 3.2.2 Classroom environment scale

The present study adapted the *Classroom Environment Scale* originally developed by Peng and Woodrow (2010). This scale encompasses three dimensions—teacher immediacy, group cohesion, and task orientation—comprising a total of 13 items. Teacher support is conceptualized as the degree to which instructors provide assistance, exhibit friendliness, and foster trust, and is measured by four items, for example, *The teacher is patient in teaching*. Group cohesion reflects the extent of mutual understanding, support, and camaraderie among students, operationalized through four items, such as *I make friends among students in this class*. Task orientation denotes students’ perceptions regarding the clarity of learning tasks, including the perceived importance and utility of these tasks, and is assessed via five items, such as *Tasks designed in this class are useful*.

All items were rated on a 5-point Likert scale, ranging from 1 (strongly disagree) to 5 (strongly agree). In the present sample, the scale demonstrated acceptable model fit indices (χ^2^/df = 1.562, CFI = 0.976, TLI = 0.972, RMSEA = 0.034, SRMR = 0.028) and good internal consistency (Cronbach’s α = 0.910). CE was modeled as a second-order latent factor, with teacher immediacy, group cohesion, and task orientation as first-order factors and their respective items as observed indicators.

#### 3.2.3 Grit scale

The *Grit Scale* developed by Duckworth and Quinn (2009) was employed in the present study. This instrument comprises two latent dimensions, perseverance of effort and consistency of interest, spanning a total of eight items. Specifically, four items are designed to assess the extent to which participants demonstrate sustained effort in their learning endeavors (e.g., *I am able to overcome significant setbacks in my studies and persist in challenging myself*), while the remaining four items evaluate the degree of stability in participants’ interests regarding their academic pursuits (e.g., *My interests in study tend to change from month to month*). The consistency of interest subscale includes reverse-worded items. All reverse-worded items were reverse-coded before analysis.

Each item is rated on a 5-point Likert scale ranging from 1 (strongly disagree) to 5 (strongly agree). All reverse-worded COI items were reverse-coded before scoring. A two-factor item-level CFA of the eight grit items showed satisfactory fit (χ^2^/df = 1.244, CFI = 0.994, TLI = 0.992, RMSEA = 0.031, SRMR = 0.036), and internal consistency was acceptable for both dimensions (POE: Cronbach’s α = 0.86; COI: Cronbach’s α = 0.82). For descriptive analyses, POE and COI were represented as separate observed subscale composites calculated from their respective four items. In the structural model, POE and COI were modeled as latent variables with their four respective items as observed indicators. Item parceling was not used.

### 3.3 Data analysis

Descriptive statistics and correlation analyses were initially performed using SPSS 26.0. Subsequently, the proposed model was assessed with AMOS 26.0. The parameter estimation process employed the maximum likelihood method, and the model’s fit was evaluated using structural equation modeling indices. In particular, an acceptable model fit was indicated by a *χ^2^/df* between 1 and 3, CFI and TLI values greater than 0.90, as well as RMSEA and SRMR values below 0.08 (Hair et al., 2019).

Before testing the structural model, confirmatory factor analyses were used to evaluate the psychometric structure of the study measures. The structural model included 32 observed indicators. The model had 528 distinct covariance moments, 330 freely estimated parameters, and 198 degrees of freedom. The item-level CFA had 323 freely estimated parameters and 205 degrees of freedom; the seven additional parameters in the structural model reflect the structural paths and covariances specified among the latent variables. The overall measurement assessment showed acceptable fit: χ^2^ = 312.45, df = 205, χ^2^/df = 1.52, CFI = 0.973, TLI = 0.969, RMSEA = 0.036, 90% CI [0.028, 0.044], SRMR = 0.029. All standardized factor loadings in the item-level CFA were significant (*p* < 0.001), ranging from 0.61 to 0.86. Composite reliability (CR) ranged from 0.84 to 0.92, and average variance extracted (AVE) ranged from 0.57 to 0.71. Discriminant validity was further supported by the Fornell-Larcker criterion and HTMT values, which ranged from 0.41 to 0.68, all below the 0.85 threshold.

Common method bias was assessed using Harman’s single-factor test and a common latent method factor analysis. Distributional assumptions were examined before the main analysis. Skewness ranged from −0.62 to 0.48, and kurtosis ranged from −0.71 to 0.93. Mardia’s multivariate normality coefficient was non-significant (*p* > 0.05), and no influential outliers were detected, supporting the use of maximum likelihood estimation.

To account for the nested structure of the data, intraclass correlations (ICCs) were calculated for the main study variables. ICCs ranged from 0.03 to 0.06, indicating minimal clustering. Cluster-robust standard errors were also examined as a robustness check, and the substantive results remained unchanged.

Finally, prospective indirect effects were evaluated using 5,000 bootstrap resamples (Hair et al., 2019). The specific indirect effect through POE and the specific indirect effect through COI were estimated separately. Bias-corrected bootstrap confidence intervals were used to assess whether each indirect effect differed from zero. Because the two indirect effects were not formally contrasted in the available analysis, statistical significance of one indirect effect and non-significance of the other is not interpreted as evidence that the two indirect effects differ from each other.

## 4 Findings

### 4.1 Assessment of common method Bias

Common method bias poses a potential risk in the collection of survey-based data. we first employed Harman’s single-factor test. The analysis revealed nine factors with eigenvalues exceeding 1, and the first factor accounted for 36.28% of the variance, which is below the recommended threshold of 40%. This result suggested that the data did not exhibit an obvious single-factor structure. To further assess common method bias, we specified a common latent method factor model. The common method factor accounted for 8.7% of the variance, and the substantive path estimates remained stable after including this factor. Taken together, these results reduce but do not rule out the possibility of common method bias.

### 4.2 Overview of classroom environment, grit, and learning engagement

Descriptive statistics and correlational analyses were performed for each variable (refer to Table 2) to provide an empirical snapshot of the classroom environment, grit, and learning engagement. The descriptive data show that participants generally rated the constructs highly, with mean scores of 3.85 for classroom environment, 3.73 for learning engagement, 3.64 for perseverance of effort, and 3.54 for consistency of interest. Furthermore, the correlational analysis indicated that learning engagement had significant positive relationships with consistency of interest (*r* = 0.44, *p* < 0.01), classroom environment (*r* = 0.39, *p* < 0.01), and perseverance of effort (*r* = 0.24, *p* < 0.01). Additionally, classroom environment was also positively correlated with both consistency of interest (*r* = 0.37, *p* < 0.01) and perseverance of effort (*r* = 0.34, *p* < 0.01).

**Table 2:** Descriptive statistics and correlation analysis of study variables.

Variable	M	SD	CE	POE	COI	LE
CE	3.85	0.76	1			
POE	3.64	0.98	0.34**	1		
COI	3.54	0.99	0.37**	0.47**	1	
LE	3.73	0.82	0.39**	0.24**	0.44**	1

**p < 0.01.

CE, classroom environment; POE, perseverance of effort; COI, consistency of interest; LE, learning engagement.

### 4.3 Results of the structural equation model

We utilized structural equation modeling (SEM) to examine both the direct and indirect routes through which classroom environment is associated with learning engagement via grit. The 32-indicator SEM demonstrated acceptable fit indices: χ^2^ = 298.76, df = 198, χ^2^/df = 1.51, CFI = 0.975, TLI = 0.971, RMSEA = 0.035, 90% CI [0.027, 0.043], and SRMR = 0.028. The standardized coefficients for the SEM are displayed in Figure 2.

**Figure 2:**
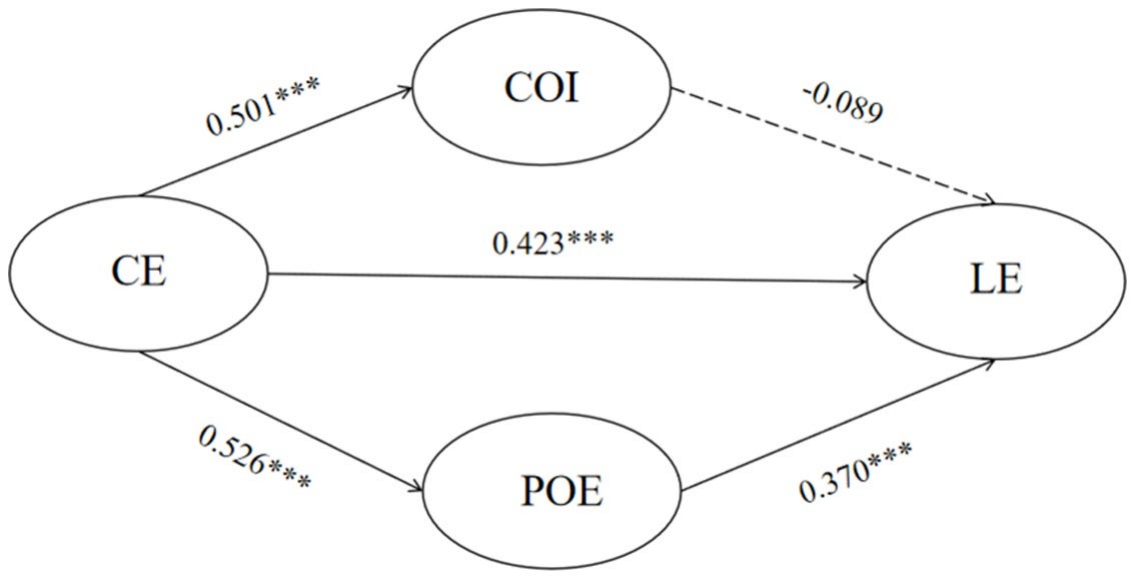
Structural equation modelling analysis. 32 indicators, df = 198; measurement indicators omitted for clarity.

Regarding the direct associations, classroom environment was significantly and positively associated with learning engagement (*β* = 0.42, *p* < 0.001), supporting H1. Perseverance of effort was also significantly and positively associated with learning engagement (*β* = 0.37, *p* < 0.001), supporting H2. In response to RQ1, the unique structural coefficient from consistency of interest to learning engagement was negative but not statistically significant (*β* = −0.09, *p* = 0.162); therefore, the model did not provide evidence for a unique COI-LE association after the other variables were entered. This conditional coefficient differs markedly from the positive zero-order correlation between COI and LE (*r* = 0.44, *p* < 0.01). Observed-score multicollinearity diagnostics calculated from the predictor correlation matrix were low (VIF: CE = 1.21, POE = 1.34, COI = 1.37), suggesting that severe conventional multicollinearity alone is unlikely to explain the sign change. The reversal may instead reflect suppression or model specification, differences between observed-score and latent-variable estimation, or method variance associated with the reverse-worded COI items. Accordingly, the negative sign is treated as model-dependent and is not interpreted as evidence that COI is harmful. Classroom environment was significantly and positively associated with both COI (β = 0.50, *p* < 0.001) and POE (β = 0.53, *p* < 0.001). The unstandardized coefficients, standard errors, and maximum-likelihood 95% confidence intervals for the structural paths are reported in Table 3.

**Table 3:** Structural model results.

Path	Unstandardized B	SE	95% CI for B (ML)	Standardized β	*p*
CE → LE	0.46	0.07	[0.32, 0.60]	0.42	<0.001
CE → POE	0.68	0.08	[0.52, 0.84]	0.53	<0.001
CE → COI	0.65	0.08	[0.49, 0.81]	0.50	<0.001
POE → LE	0.31	0.06	[0.19, 0.43]	0.37	<0.001
COI → LE	−0.07	0.05	[−0.17, 0.03]	−0.09	0.162

***p < 0.001.

The 95% confidence intervals in this table are Wald-type maximum-likelihood intervals for the unstandardized coefficients (B), not bootstrap intervals. R^2^ for LE = 0.41; R^2^ for POE = 0.28; R^2^ for COI = 0.25. Covariance between POE and COI = 0.21, SE = 0.04. Observed-score VIFs based on the CE, POE, and COI correlation matrix were 1.21, 1.34, and 1.37, respectively.

As an observed-score sensitivity check, we reconstructed standardized regression coefficients from the rounded correlation matrix in Table 2 (*N* = 468). In a POE-only model, POE was positively associated with learning engagement (*β* = 0.24, *p* < 0.001). In a COI-only model, COI was positively associated with learning engagement (*β* = 0.44, *p* < 0.001). When POE and COI were entered simultaneously, COI remained positive and significant (*β* = 0.42, *p* < 0.001), whereas POE was small and nonsignificant (*β* = 0.04, *p* = 0.366). When classroom environment, POE, and COI were entered simultaneously, classroom environment remained positive (*β* = 0.27, *p* < 0.001), COI remained positive (*β* = 0.35, *p* < 0.001), and POE was nonsignificant (*β* = −0.01, *p* = 0.762). Thus, the negative COI coefficient in the latent structural model was not reproduced in the observed-score sensitivity analyses, suggesting that the sign reversal is model-dependent rather than a simple consequence of conventional multicollinearity. Because these coefficients were reconstructed from rounded published correlations rather than estimated from the raw dataset, they are presented as approximate robustness diagnostics and do not substitute for refitting the requested alternative SEM specifications.

Bias-corrected bootstrap sampling (5,000 resamples) was used to evaluate the prospective indirect effects (Table 4). The specific indirect effect from T1 classroom environment to T3 learning engagement through T2 perseverance of effort was statistically significant: unstandardized B = 0.211, bootstrap SE = 0.065, BC 95% CI [0.07, 0.32], bootstrap *p* = 0.003; standardized *β* = 0.20, BC 95% CI [0.07, 0.32]. In contrast, the specific indirect effect through T2 consistency of interest was not statistically significant: unstandardized B = −0.05, bootstrap SE = 0.04, BC 95% CI [−0.13, 0.04], bootstrap *p* = 0.28; standardized *β* = −0.04, BC 95% CI [−0.13, 0.03]. The BC 95% CI for the COI indirect effect included zero. Because the two specific indirect effects were not formally contrasted, the significant POE indirect effect and the non-significant COI indirect effect should not be interpreted as evidence that the two indirect pathways differ statistically. The direct association between classroom environment and learning engagement remained statistically significant, B = 0.46, SE = 0.07, *p* < 0.001, standardized *β* = 0.42.

**Table 4:** Unstandardized and standardized direct, indirect, and total effects from the prospective indirect-effects model.

Effect	Path	Unstandardized B	Bootstrap SE	BC 95% CI for B	Bootstrap *p*	Standardized β	BC bootstrap 95% CI for β
Total	CE → LE	0.63	0.07	[0.49,0.77]	<0.001	0.57	[0.42, 0.67]
Direct	CE → LE	0.46	0.07	[0.32,0.60]	<0.001	0.42	[0.26, 0.57]
Indirect	CE → POE → LE	0.21	0.07	[0.07,0.32]	0.003	0.20	[0.07, 0.32]
Indirect	CE → COI → LE	−0.05	0.04	[−0.13,0.04]	0.279	−0.04	[−0.13,0.03]

## 5 Discussion

### 5.1 The direct relationship between classroom environment and learning engagement

The positive association between classroom environment and learning engagement in MHEC reinforces a central tenet of positive psychology: that organizational structures characterized by support, cohesion, and meaningful tasks are associated with positive behavioral outcomes (Seligman and Csikszentmihalyi, 2000). Unlike general academic courses, MHEC requires students not only to acquire knowledge but also to internalize psychological competencies such as emotional regulation and stress management, a process that demands sustained, high-quality engagement (Gao and Zong, 2017; Tan and Ma, 2020). The three dimensions we examined, teacher immediacy, group cohesion, and task orientation, each tap into distinct mechanisms posited by self-determination theory (Joe et al., 2017). Teacher immediacy satisfies the need for relatedness and competence by providing clear guidance and emotional support; group cohesion fulfills belongingness needs; and task orientation, especially when tasks are authentic and challenging, supports autonomy and competence. When these needs are met, students are more likely to exhibit behavioral, emotional, and cognitive engagement (Fredricks et al., 2004). This study thus provides domain-specific evidence that a well-designed classroom environment may serve as a crucial external resource that supports rather than merely invites engagement.

Yet the relationship is not unconditional. Prior research has shown that students’ responses to the same classroom environment can vary significantly, with some thriving under pressure and others disengaging in relaxed settings (Gao, 2016). Our finding that classroom environment was directly associated with learning engagement suggests that environmental factors may have a robust, independent association with engagement. However, the magnitude of this direct association (*β* = 0.423) leaves room for additional indirect pathways, which we examine below. A deeper theoretical implication is that classroom environment in MHEC may operate not only as a direct contextual correlate but also through individual characteristics. This perspective shifts the focus from asking whether classroom environment is associated with engagement to examining potential intervening pathways, a question addressed here through prospective indirect-effect analysis.

The study also contributes methodologically by employing a three-wave time-lagged design that temporally separates the measurement of environment, grit, and engagement. This reduces the risk of recall bias and common method variance, strengthening the interpretability of the direct association as a prospective association. Nevertheless, because we did not manipulate the environment, we refrain from causal language.

### 5.2 The direct relationship between grit and learning engagement

The dimension-specific pattern observed in the structural model, with POE positively associated with learning engagement and the unique COI coefficient not statistically significant, supports the value of examining the two grit dimensions separately rather than combining them into a single score. Perseverance of effort has been consistently linked to academic outcomes across diverse contexts (Datu et al., 2018; Teimouri et al., 2022), and meta-analytic evidence suggests that POE often shows stronger achievement-related associations than COI (Credé et al., 2017). In MHEC, which is typically compulsory for Chinese university students, sustained effort may be especially relevant because students must continue engaging even when their interest fluctuates. This interpretation is consistent with goal orientation theory (Wolgast and Keller-Schneider, 2024): students high in perseverance of effort may be more likely to persist with challenging or emotionally demanding material.

The COI result requires a more cautious interpretation. At the zero-order level, COI was positively correlated with learning engagement, whereas its unique structural coefficient became negative and non-significant after the full SEM was specified. Because the observed-score VIFs were low, severe conventional multicollinearity does not appear to be a sufficient explanation for this reversal. The observed-score sensitivity regressions also did not reproduce the negative COI coefficient: COI remained positive when entered alone, together with POE, and together with CE and POE. This discrepancy points more strongly to a model-specification or measurement-level explanation, including differences between latent-variable and observed-score analyses, suppression induced by the latent specification, or method effects associated with reverse-worded COI items. The present result therefore should not be interpreted as showing that consistency of interest is negatively related to engagement. Rather, it indicates that the positive bivariate association did not translate into a statistically reliable unique association in the specified latent structural model. Raw-data alternative SEM analyses remain necessary to determine which specification is most robust.

Importantly, the non-significant unique COI coefficient should not be interpreted as evidence that passion or stable interest is irrelevant. The finding is conditional on the present model and sample. It nevertheless underscores the importance of treating grit as a multidimensional construct and reporting POE and COI separately rather than relying only on a total grit score (Fernández et al., 2020). In the present data, combining the two dimensions could obscure their different conditional associations with learning engagement.

### 5.3 Prospective indirect associations through grit

A statistically significant prospective indirect association was observed from T1 classroom environment to T3 learning engagement through T2 perseverance of effort. This pattern is consistent with the proposition that a supportive classroom environment may be associated with greater perseverance by satisfying students’ needs for autonomy, competence, and relatedness (Ryan and Deci, 2000), and that perseverance is in turn associated with later engagement. The result is also compatible with prior work linking classroom climate, grit, and academic outcomes (Wei et al., 2019; O'Neal et al., 2018). However, because POE and learning engagement were not measured at baseline and each construct was assessed only once, the observed indirect effect should be interpreted as a prospective statistical association rather than as evidence that classroom environment caused a change in POE which then caused a change in engagement.

The specific indirect effect through COI was not statistically significant. Although classroom environment was positively associated with COI, the unique COI-to-engagement path in the full model was not significant. One possible interpretation is that stable interest may be less central in a compulsory course in which interest is still developing, but this explanation is *post hoc* because initial interest, perceived compulsory status, and motivational regulation were not measured. In addition, the two specific indirect effects were not formally contrasted. Therefore, the pattern should be described as one significant and one non-significant indirect estimate, not as statistical evidence that the POE and COI indirect effects differ from each other.

The direct association between classroom environment and learning engagement remained statistically significant after the two grit dimensions were included. This suggests that other unmeasured processes may also link classroom conditions to later engagement, such as positive emotions (MacIntyre, 2016) or academic self-efficacy (Bandura, 2002). Practically, the findings support attention to both classroom conditions and students’ sustained effort. Recommendations concerning COI should be more tentative: the current data do not show a reliable unique or indirect COI association in the full model, but they also do not show that COI is detrimental. Interpretations involving compulsory-course status and motivational internalization remain *post hoc* and require direct testing.

## 6 Conclusion

### 6.1 Main findings

Grounded in the Three Pillars Theory of Positive Psychology and integrated with Self-Determination Theory and Social Cognitive Theory, this study used a three-wave time-lagged survey to examine temporally ordered prospective associations among classroom environment, the two dimensions of grit, and learning engagement among first-year students enrolled in MHEC at one university in East China. Classroom environment and POE were positively associated with learning engagement. The unique COI-to-engagement coefficient was not statistically significant in the full structural model, despite a positive zero-order correlation. Bootstrap analyses indicated a significant prospective indirect association through POE and a non-significant indirect association through COI. Because baseline levels were not controlled, each construct was measured only once, and the two specific indirect effects were not formally contrasted, these findings should not be interpreted as causal mediation, change in grit over time, or evidence that the two indirect pathways differ statistically.

### 6.2 Research significance

Theoretically, this study extends the classroom environment-learning engagement framework to the under-researched domain of mental health education and demonstrates the value of separating POE and COI. The results provide preliminary evidence of dimension-specific conditional associations in a compulsory-course context, but they do not justify a claim that one grit dimension has a significantly stronger indirect effect than the other. The non-significant unique and indirect COI estimates raise the possibility that compulsory course status or developing interest may constitute boundary conditions, but these explanations require direct measurement and replication. The observed sign change for COI also indicates that conclusions about this dimension are sensitive to model specification and should be tested in alternative models.

Practically, this study suggests that educators should create supportive classroom environments by enhancing teacher immediacy, fostering group cohesion, and designing clear, meaningful tasks. The positive POE pathway also suggests potential value in supporting students’ sustained effort through appropriately challenging tasks, goal segmentation, feedback, progress monitoring, and strategic support during difficulty. Recommendations regarding COI should remain cautious. Because its unique and indirect associations were not statistically significant in the full model, the present data do not support interventions that focus on increasing consistency of interest alone; future work should test whether situational interest and motivational internalization are more relevant mechanisms in compulsory MHEC.

### 6.3 Limitations and future directions

This study has several limitations. First, although the three-wave time-lagged design temporally separated classroom environment, grit, and learning engagement, each construct was measured only once. Baseline levels of POE, COI, and learning engagement were not controlled, and the design cannot estimate autoregressive stability, within-person change, or change in grit over time. The indirect effects should therefore be interpreted as prospective indirect associations rather than as causal or fully longitudinal mediation. Future studies should use repeated measurement of the predictor, mediators, and outcome and apply cross-lagged panel, random-intercept cross-lagged, latent-change, or experimental designs where appropriate. Second, although POE and COI were modeled as latent variables with their four respective items as indicators, the structural model was complex, with 32 observed indicators, 330 freely estimated parameters, and 198 degrees of freedom. Third, the convenience sample from a single university in East China limits external validity; future studies should include diverse regions and institution types. Fourth, self-report measures may introduce method bias, and potential confounders such as prior mental health status and socioeconomic background were not controlled. Perceived compulsory status, initial interest, and motivational internalization were also not measured, so related interpretations are *post hoc*. Finally, the marked difference between the positive zero-order COI-LE correlation and the non-significant negative structural coefficient requires additional robustness analysis, including alternative SEM specifications and examination of possible reverse-wording method effects. A formal bootstrap contrast between the POE and COI specific indirect effects is also needed before concluding that the pathways differ statistically.

## Data Availability

The raw data supporting the conclusions of this article will be made available by the authors, without undue reservation.

## Footnotes

1
https://www.wjx.cn/
